# Medical Opinion and Movement

**Published:** 1910-03-19

**Authors:** 


### Medical Opinion and Movement.

### Arthritis Deformans and Syphilis.

If the Wassermann reaction can be accepted as absolutely specific for syphilis or a so-called syphilitic taint, it promises to offer a new means of research into all kinds of morbid conditions of doubtful pathological origin, some of which at any rate may eventually prove to have a causal connection with syphilitic disease, congenital or otherwise. One of the latest investigations of this kind is that of Dr. J. Heckman, of New York. He reports in the *Medical Record* that he has examined four cases of monarticular and eleven cases of the polyarticular form of arthritis deformans, and has found a positive Wassermann reaction in all four cases of the monarticular type, and in most of the polyarticular forms of the disease. He therefore believes that syphilis plays a large rôle in the ætiology of this disease. In all the monarticular cases there was a history of trauma, and he suggests that in these cases the trauma is the exciting and syphilis the underlying cause. In the polyarticular cases he supposes that a rheumatic diathesis is the exciting cause which determines this particular manifestation of syphilitic disease. It is probably by no means the whole truth in this very complex question of the ætiology of arthritis deformans, or rheumatoid arthritis, but this line of investigation certainly deserves further attention.

### A New Theory of Colour.

The reason why we perceive the differences and harmonies in colour has not yet received a sufficiently satisfactory explanation. The theory of Helmholz, which makes these differences depend upon the wave-lengths of the different coloured rays of light, is not without objections. In order to bring the theory into accord with spectral analysis it compels the adoption of blue, yellow, and red as the fundamental colours instead of the more natural scale, hitherto accepted by painters and physiologists, of violet-orange-red. Dr. W. Nicati, of Marseilles, following this line of thought, has developed an ingenious hypothesis to explain these discrepancies. By a series of experimental observations he has measured the degree of sensibility to light of the whole visual field and also of the central visual area, and has in this way established a close parallel between the visual and auditory senses. As in acoustics, so in vision he distinguishes three fundamental harmonies—the harmonies of intervals, the harmonies of combination or accord, and the harmonies of contrast or complement. The colours are the harmonic intervals comparable to the seconds, thirds, fifths, and other intervals of music, and he arranges them in a scale of eight intervals corresponding to the octave scale of music, thus—white, violet, blue, green, yellow, orange, crimson, and red. He gives the fractions representing the degrees of sensibility of these different colours, ascertained by experiment, by which they naturally fall in place in this order. The harmonic combinations are the mixed colours, and the harmonic contrasts are the complementary colours. Such is briefly the theory, and it would appear to conform to facts in a very pretty manner. Thus in the case of the fundamental colours, blue, yellow, red, we have an exact equivalent of the major chord of music—third, fifth, and octave.

### A New Sign for Tetany.

At a meeting of the Medical Society of Vienna Dr. H. Schlesinger brought forward a new sign for tetany, closely analogous to that of Trousseau, which he has discovered, and which he terms "the sign of the leg." If the thigh is flexed at the hip joint, at the end of a minute or two painful spasms occur in the muscles of the flexed limb. The leg is drawn out in a position of extension and the foot in a position of exaggerated supination, while the toes are unaffected. As in the case of Trousseau's sign, the spasm is limited to the muscles of the flexed limb, and ceases as soon as the limb assumes a normal position. The essential condition for the appearance of the spasm is the flexion of the thigh. It occurs also in both limbs if the patient is made to sit up in bed without flexing the knees, or, with the patient standing, by bending the body at the hips. Pressure on the sciatic nerve does not produce the spasm, and it does not appear to be due to any circulatory disturbance. It is probably of a reflex nature. Dr. Schlesinger has not found the sign present in any other affection. Dr. Gottlieb also stated that he had obtained the phenomenon in an infant aged one and a-half years, suffering from rickets, in whom the sign of Trousseau and also that of Chvosteck were present.

### Rectal Saline Irrigation in Typhoid.

The good results obtained by continual irrigation of the rectum with normal saline solution in surgical cases induced Dr. D. Riesman of Philadelphia to try this method of treatment in cases of typhoid fever, in the hope that in this way one might facilitate the elimination of toxines from the system. For this purpose the author uses the ordinary physiological solution of sodium chloride without addition of calcium chloride. He has the apparatus working all day, with short intervals, so that there is a continuous instillation of the fluid into the rectum, but does not allow it to continue through the night. In this way 2½ to 4½ litres of fluid may be introduced into the system during the day. Dr. Riesman has employed the method in twelve cases of typhoid fever in the Jewish Hospital in Philadelphia, several of which were of a serious nature. All the patients recovered. The good effects of the treatment were seen in the increased diuresis and in the marked improvement in the general nervous symptoms. The idea naturally occurs, however, that the introduction of so much fluid into the system might easily increase the tendency to hypostatic congestion of the lungs, especially in prolonged cases, in which the heart's action becomes enfeebled, or in cases in which the kidneys may not be functioning efficiently, and in which the fluid would tend to accumulate in the tissue. These contingencies should therefore be borne in mind, and considerable attention exercised in the control of the treatment.

### Tracheal Pushing.

Tracheal tugging is a sign of aortic aneurysm with which every student is familiar; it is one of those aids to diagnosis which by some inherent touch of the dramatic appeal strongly to human nature, though as a matter of fact the proportion of sufferers from this condition who exhibit it is not enormous. Recently two observers have independently described the reverse phenomenon of tracheal pushing; that is, a jerking of the larynx and trachea upwards instead of downwards with each beat of the heart. One of these cases was an example of that extremely rare condition, thoracic aneurysm in a child. The patient was exhibited not long ago at one of the London societies. The other case takes precedence in point of time, for the details of it were published last October in the *Bulletin de la Société Medicale des Hopitaux* by Dr. Hirtz. The tracheal tug is generally ascribed to pressure on the bronchus of an aneurysm situated on the concavity of the aortic arch. This latter author suggests for the explanation of the reversed sign that it may be due to pulsation in an aneurysm of the convexity of the arch, but even then it is rather difficult to comprehend against what structure exactly the pressure is supposed to be applied.

### The Best Salt of Quinine.

In a lecture on the treatment of malaria published in the *Medical Magazine* Dr. F. M. Sandwith emphasises the importance of selecting the best of the 20 different salts of quinine which are upon the market. The most popular by a long way is the sulphate, but this is really, he says, that which he would choose last of all. The sulphate of quinine is soluble only in 800 parts of water, unless acid is added; constipation and dyspepsia are readily excited by prescribing it thus. If given as pill or tablet, it often passes in the fæces undissolved. Much the best salt, in the author's opinion, is the bihydrochloride, which is soluble in two parts of water, and can be used, if necessary, for intramuscular injection. The only drawback is the cost, which is twice that of the sulphate. Next stands in popular estimation the hydrobromide, which is supposed to have less tendency to cause cinchonism; this belief is quite erroneous, Dr. Sandwith says. The hydrochloride is also popular, and its price is about midway between those of the sulphate and bihydrochloride; in efficiency and solubility it is inferior to the latter. Of the bisulphate he has almost as much to say in favour as of the bihydrochloride; the former of these two has been adopted for universal use in the British Army. As to administration, Dr. Sandwith favours intramuscular or intravenous injection in urgent cases; and solutions or cachets if given by the mouth. Subcutaneous injection is condemned strongly, and so are pills and tablets. Those who prefer Warburg's tincture, he declares, are they who have not learned how and when to give quinine.

### Fracture of the Base of the Skull.

In the *Revue de Chirurgie* Dr. E. Vincent proposes that operative treatment by trephining should be adopted for all cases of fracture of the base of the skull, on the ground that the frequency of fatal suppurative meningitis can thus be diminished. Whenever, in fact, there is any communication between the meningeal space and the ear or pharynx, he advises drainage of the cerebro-spinal fluid through an independent opening made somewhere in the vault of the skull and as near as possible to the seat of fracture of the base. At the same time that the dura mater is opened for this purpose it is possible to evacuate any clot which may have collected. A drain is put into the wound in direct contact with the surface of the brain, and the skin sutured except where the tube emerges. This drainage is maintained for a fortnight, after which time the likelihood of any infection through the cracked base is almost abolished. The operation should be performed at the earliest possible moment. Two cases are described in which this was carried out; but, unfortunately for the author's argument, in one of them infection reached the brain through the operation wound, with a fatal issue. The frequency of suppurative meningitis after fracture of the base is probably over-estimated by Dr. Vincent, though it must be allowed not to be at all unfamiliar: the heroic remedy proposed he has still to prove no worse than the evils which he would avoid.

### Preparation for Vaginal Operations.

The practice of preparing the cutaneous site of a proposed operation by painting it with a solution of iodine, with or without acetone or alcohol in addition, has been growing steadily in favour in this country since its introduction abroad by Grossich and others. This method has previously received notice in these columns, and it now appears that the use of iodine has been extended to the cleansing of the vagina preliminary to operations conducted by that route. Dr. Chevrier describes in the *Gazette des Hopitaux* the method which he finds reliable. He exposes the cervix uteri by holding apart the vaginal walls with two aseptic specula. The mucous membrane is then mopped over very carefully with swabs dipped in ether, and by altering the position of the specula the whole surface is thus treated. Then a similar process is repeated, using tincture of iodine instead of ether; no liquid iodine solution is to be allowed to remain in the vagina. The results are said to be much superior to those of the usual systems of douching; and as the whole preparation is conducted under anæsthesia just before the operation begins, the advantages from the point of view of the patient's comfort are obvious.

### General Anæsthesia by Means of X-Rays.

In the *New Zealand Medical Journal*, under the above heading, Dr. J. P. Hastings, hon. anæsthetist to the Dunedin Hospital, describes some original experiments made by him on the guinea-pig. After reading the work of Leduc, of Nantes, on the production of general anæsthesia in animals by means of the interrupted electrical current, he was struck with the idea that similar effects might be produced by the *x*-rays. The tube used was a hard Crookes', the strength of current in the coil 5-6 ampères, the length of spark 12 inches, and the strength of current in the tube itself 2-3 milliampères. The following are the author's suggestions with regard to the best position in which to place the animal: "Draw an imaginary line at right angles to the surface of the anti-kathode; on this line take a point one inch from the surface of the Crookes' tube; place the animal to be anæsthetised so that the imaginary line cuts the back of the skull at the point indicated." Anæsthesia is complete in two to three minutes after the commencement of the exposure, the time varying slightly in different animals. The length of time varies inversely as the strength of the current and the distance of the animal from the tube. Previous to the onset of anæsthesia there is a stage of excitement during which the animal struggles convulsively. The struggles gradually decrease until unconsciousness and complete relaxation are present. The breathing and heart-beats lessen in intensity. Anæsthesia lasts five to seven minutes, during which there is complete insensibility to external stimuli. As the effects pass off the heart commences to beat more strongly and the respirations become deeper. Consciousness gradually returns, the animal stands up, and moves about in a dazed fashion. Finally it runs about as well as ever. There are no after-effects, such as nausea, and no apparent impairment of intelligence. The results have been approximately the same in all cases, and no death has occurred. The author thinks that radium emanations may have a similar effect of inducing anæsthesia, in that the gamma-rays of radium are comparable to the *x*-rays, and resemble the latter in penetrative power. He is uncertain whether the subject of *x*-rays anæsthesia will ultimately prove of more than academic interest, and would welcome further experiments with a view to elucidating the point. He is at present investigating the possibilities of blue light as a producer of anæsthesia.

### The Effects of Formol.

Formol is often added fraudulently to milk as a preservative. With the object of ascertaining the effects produced on the human organism by the repeated ingestion of small doses of this drug, M. Willay, according to *l'Hygiène de la viande et du lait*, has persuaded a dozen healthy men to submit themselves to the following experiment. Each of them was made to take daily from 1½ to 3 grains of formol until the total quantity of the drug absorbed by each amounted to 40 grains. The drug was taken dissolved in water or in milk. The following effects were noticed by the author. For the first ten days no symptoms were complained of, but later gastric and intestinal pain, cramps, nausea and vomiting made their appearance. In four cases well-marked eruptions appeared on the skin of the chest and thighs, while several patients complained of burning sensations in the throat. The urine increased in amount and the fæces became watery. The patients lost on an average from 6 to 12 oz. in weight daily. It would therefore seem that the introduction of formic aldehyde into food is dangerous to public health, and particularly objectionable when added to milk, which eventually may be consumed by infants and invalids.

### Excretion of Arsenic in Menstruation.

Hertoghe recently has reported to the Belgian Royal Academy of Medicine the researches of Vayas and Gauthier into the question of the elimination of arsenic by the menstrual blood. From the experiments carried out by these workers he comes to the following conclusions: (1) That the menstrual blood, as blood, is not the sole agent in the elimination of the drug; (2) that the pre-menstrual pre-hæmorrhagic secretions are already impregnated with arsenic; (3) that the post-hæmorrhagic secretions contain none of the drug; (4) that arsenical elimination does not occur in the course of every catamenia, but at irregular intervals which may perhaps correspond to periods of possible fecundation. The author insists on the interest of the results of chemical analysis of the sexual secretions in various morbid states such as anæmia, chlorosis, leukæmia, certain forms of sterility, diseases of the ovaries and uterus, and the various intoxications of pregnancy.

### Chlamydozoa in Ophthalmia Neonatorum.

Halberstaedter and Von Prowazek, in the *Berlin Klin. Woch.*, report the results of experiments carried out by themselves with the object of determining whether the intracellular bodies, termed chlamydozoa, found in cases of trachoma are specific for this disease, or whether they also are met with, as some authors maintain, in cases of gonorrhœal ophthalmia. In order to investigate the facts they have carefully made examinations of a large number of cases, and the results that they have obtained are interesting and ought to prove stimulative to future investigators. In the discharges and scrapings obtained from a number of cases of urethritis and vaginitis, both acute and chronic, the authors were unable to discover these chlamydozoa. A series of cases of ophthalmia neonatorum, in the discharges from which gonococci were found, also failed to show these intracellular bodies. Five cases of this disease, however, clinically indistinguishable from the rest, but in which no gonococci could be found either in smears or in cultures, showed the presence of chlamydozoa in large numbers. These bodies were also met with in the vaginal discharge obtained from the mother of one of the cases. From these facts the authors conclude that there is a form of ophthalmia of the new-born due, not to the gonococcus, but to a parasite indistinguishable morphologically from the trachoma bodies. It is not certain whether the bodies found in the two diseases are identical, but in both they would seem to be chlamydozoa.

